# Human β-defensin-3 reduces excessive autophagy in intestinal epithelial cells and in experimental necrotizing enterocolitis

**DOI:** 10.1038/s41598-019-56535-3

**Published:** 2019-12-27

**Authors:** Liping Chen, Zhibao Lv, Zhimei Gao, Guijie Ge, Xueli Wang, Junmei Zhou, Qingfeng Sheng

**Affiliations:** 1 Department of General Surgery, Shanghai Children’s Hospital, Shanghai Jiao Tong University, Shanghai, 200062 China; 2 Department of Central Laboratory, Children’s Hospital of Shanghai, Shanghai Jiao Tong University, Shanghai, 200062 China; 3 Department of Pathology, Children’s Hospital of Shanghai, Shanghai Jiao Tong University, Shanghai, 200062 China

**Keywords:** Infant necrotizing enterocolitis, Infant necrotizing enterocolitis

## Abstract

Necrotizing enterocolitis (NEC) is a leading cause of mortality in preterm newborns. Intestinal barrier dysfunction is one key event in NEC pathogenesis. Human β-defensin-3 (hBD3), one member of cationic host defence peptides, was reported to reduce the development of necrotizing enterocolitis in a neonatal rat model. And autophagy was induced in the intestine of human and animals with NEC. We hypothesized that regulation of autophagy might play a critical role in hBD3-mediated protection against NEC injury. Autophagy activity was evaluated both in intestinal epithelial cells and in NEC models. Newborn Sprague-Dawley rats were divided randomly into four groups: Control + NS, Control + rapamycin, NEC + NS, and NEC + hBD3. Body weight, histological score, survival time, enterocyte migration and mucosal barrier were recorded. Our results showed that hBD3 pretreatment could effectively inhibit autophagy activity in cultured IEC-6 and Caco2 enterocytes, and CXCR4 might be involved in hBD3-mediated autophagy suppression. Moreover, hBD3-induced inhibition of autophagy significantly promoted the intestinal epithelial cell migration by wound healing assay and transwell migration assay. In the rat model of NEC, hBD3 could noticeably reduce the expression of autophagy-activated proteins, down-regulate the expression of inflammatory mediators, and promote the mucosal integrity. Our data suggest an additional role of hBD3-mediated protection against intestinal mucosal injury: inhibition of over-activated autophagy in enterocytes.

## Introduction

Necrotizing enterocolitis (NEC) is a digestive system disease which seriously threatens newborns as a result of multiple factors, such as premature birth, intestinal dysbacteriosis, formula feeding, and genetic predisposition^1,2^. Despite advancements in neonatal medicine, the mortality associated with NEC still ranges from 20% to 30%^3^. Even though many risk factors have been proved to be relevant^4,5^, the exact etiology and pathogenesis of NEC remain unknown^6^. Intestinal barrier dysfunction in premature newborns is one key cause leading to the onset of NEC.

Autophagy is an intracellular degrading and recycling pathway of proteins and organelles that relies on lysosomes, and is involved in many important biological processes, such as cell survival, cytoskeleton remodeling, antigen presentation, etc.^7,8^. Recent evidence has shown that uncontrolled activation of autophagy has been identified as a risk factor for the development of NEC^9^. Excessive activation of autophagy could damage intestinal barrier and increase permeability through degradation of tight junction protein such as claudin-2^10^. Moreover, rapamycin (a classic autophagy inducer) has been confirmed to significantly inhibit the migration of several cell types including intestinal epithelial cells both *in vitro* and *in vivo*^11^, which suggests that autophagy participates in the repair process of intestinal mucosal barrier and plays an important role in maintaining the integrity of intestinal mucosal barrier.

Human beta-defensin-3 (hBD3, OMIM: 606611), first detected in lesion tissues of psoriatic patients in 2001, is a cationic antibacterial peptide with antimicrobial activity and immune modulation linking innate and adaptive immunity^12,13^. Jenke *et al*.^14^ reported low beta defensin expression in severe NEC. In addition, we have previously reported that hBD3 treatment can significantly induce intestinal epithelial cell migration and reduce the severity and mortality of NEC model in neonatal rats^15^. However, the underlying mechanism has not been elucidated clearly.

We now hypothesized that regulation of autophagy played a critical role in hBD3-mediated protection against NEC injury. Consequently, the present study was designed to shed light on the effect of hBD3 on autophagy both in intestinal epithelial cells and in experimental NEC model.

## Results

### Pharmacologic induction of autophagy in intestinal epithelial cells

**Figure 1:**
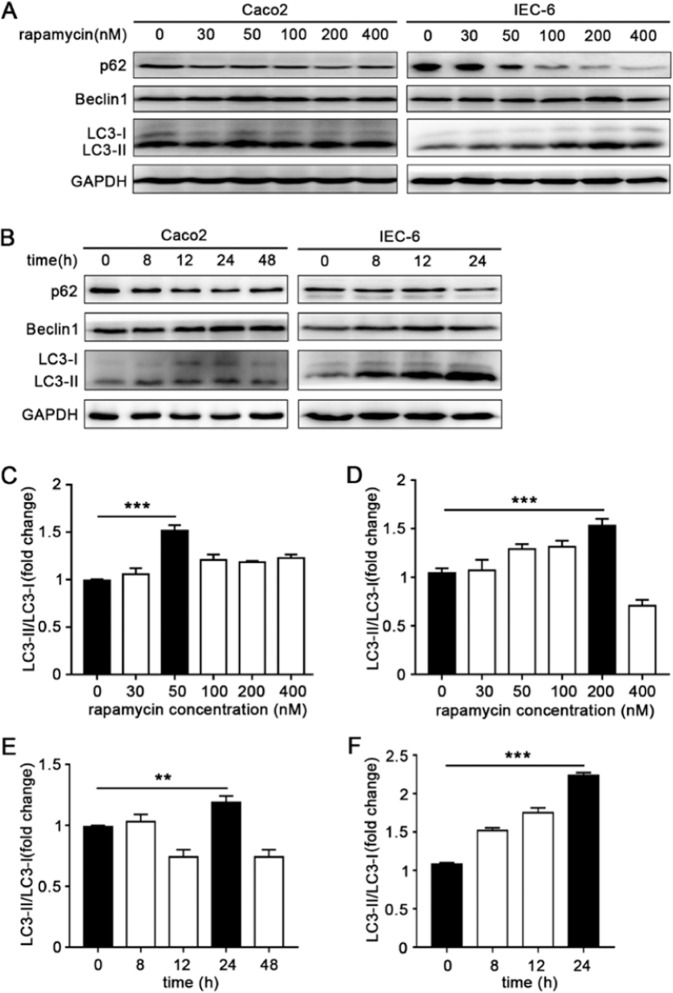
Induction of autophagy in intestinal epithelial cells. The optimal concentration (**A**) and time (**B**) of rapamycin on Caco2 and IEC-6 were respectively detected. Cell lysates were prepared and analyzed by western blotting for the expression of autophagy related proteins LC3, p62 and Beclin1. GAPDH was used as a normalization control. The LC3II/LC3I ratio of Caco2 increased significantly after 24 h incubation with 50 nM rapamycin (**C**,**E**), accordingly, 24 h treatment with 200 nM rapamycin could successfully induce autophagy of IEC-6 (**D**,**F**). Data was presented as mean ± SD for three independent experiments. **p < 0.01, ***p < 0.001.

In order to detect the optimal concentration and incubation period, intestinal epithelial cells IEC-6 and Caco2 were respectively incubated with rapamycin (a classic autophagy inducer) for the indicated concentrations and periods over a broad range. After the rapamycin administration, the conversion of microtubule-associated protein 1 light chain 3-I (LC3-I) to LC3-II, as the hallmark of autophagy, was increased both in Caco2 and IEC-6 cells. Beclin1, another essential protein as a way to monitor autophagy, was also increased after rapamycin treatment. In addition, p62 (also known as sequestosome-1), which serves as a link between LC3 and ubiquitinated substrates, was decreased in rapamycin-treated cells. According to the expression of autophagy-related proteins, the autophagy flux in Caco2 increased significantly after being incubated with 50 nM rapamycin for 24 h (Fig. 1A–C,E). Accordingly, the autophagy in IEC-6 was effectively induced by rapamycin at the concentration of 200 nM for 24 h (Fig. 1A,B,D,F).

### hBD3 inhibited autophagy in intestinal epithelial cells

**Figure 2:**
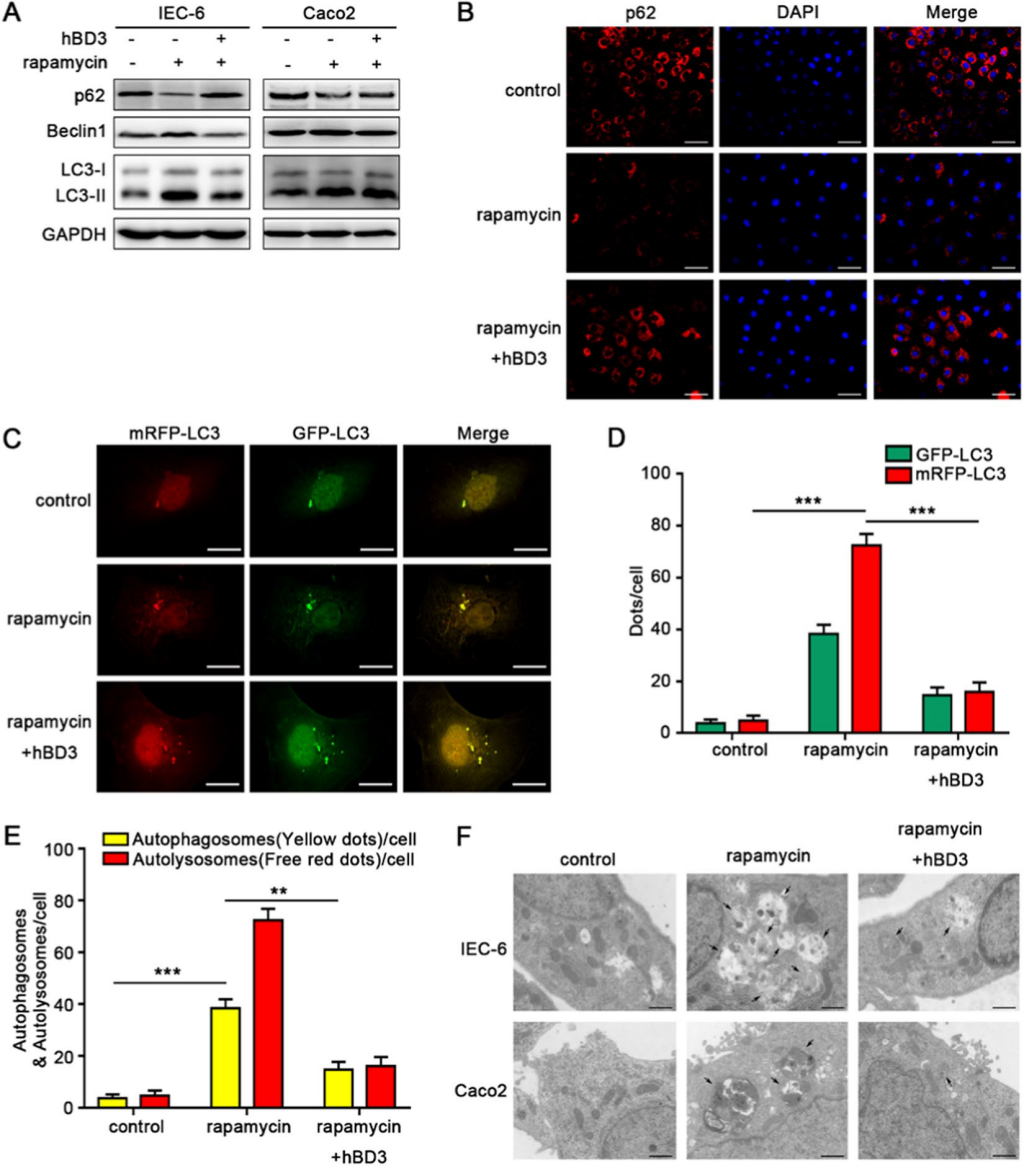
hBD3 inhibited autophagy in intestinal epithelial cells. The intestinal epithelial cells were respectively incubated with rapamycin (200 nM for IEC-6 or 50 nM for Caco2, 24 h) or hBD3 (5 μg/ml, 12 h) as indicated. The expression of p62, Beclin1 and LC3 in each group was determined by western blotting (**A**), and p62 levels of IEC-6 were also assessed by immunostaining (**B**, scale bar, 50 μm). IEC-6 cells were infected with Ad-mRFP-GFP-LC3(MOI = 1000) for 48 h and photographed with the confocal microscopy. Representative mRFP-LC3, GFP-LC3 and merge images were shown (**C**, scale bar, 20 μm). GFP-LC3 dots and mRFP-LC3 dots per cell were respectively counted (**D**) the number of yellow (autophagosome) and free red dots (autolysosomes) per cell was quantified. (**E**) The ultrastructure of IEC-6 and Caco2 cells was presented by TEM (**F**, arrows indicated degradative autophagic vacuoles (AVd), scale bar, 1 μm). Data was expressed as mean ± SD of three independent experiments. Abbreviations: hBD3, human beta defension-3; MOI, multiplicity of infection; TEM, transmission electron microscopy. **p < 0.01, ***p < 0.001.

**Figure 3:**
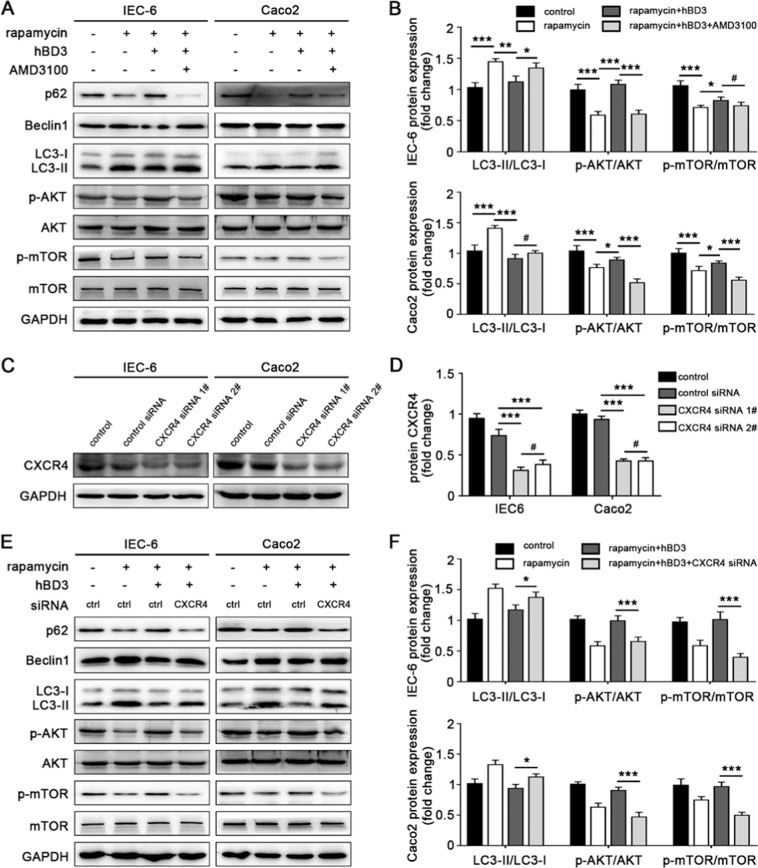
CXCR4 signaling pathway was involved in hBD3-mediated autophagy suppression. Intestinal epithelial cells IEC-6 and Caco2 were incubated with CXCR4 inhibitor AMD3100 (10 μM, 24 h), and further treated with rapamycin (200 nM for IEC-6 or 50 Nm for Caco2, 24 h) and hBD3 (5 μg/ml, 12 h) as indicated. The expression of p62, Beclin1, LC3, p-mTOR and p-AKT in each group was assessed by western blotting (**A**) the LC3II/LC3I ratio and relative expression of p-mTOR and p-AKT were analyzed (**B**) with total mTOR and AKT as a loading control. CXCR4 knockdown efficiency was monitored (**C**) and analyzed (**D**) in IEC-6 and Caco2, respectively. After being incubated with CXCR4 siRNA for 36 h and treated with rapamycin and hBD3 mentioned above, cells in each group were lysed and subjected to western blotting using antibodies against p62, Beclin1, LC3, p-mTOR and p-AKT. (**E**) The LC3II/LC3I ratio and relative expression of p-mTOR and p-AKT were also analyzed (**F**) with the same methods referred to above. Data was displayed as mean ± SD from three independent experiments. *p < 0.05, **p < 0.01, ***p < 0.001, ^#^p > 0.05.

Wu *et al*. reported that beta-defensin-3 could suppress autophagy in macrophages^16^. Thus, we studied the effects of hBD3 on the autophagic process in the intestinal epithelial cells. The expression of Beclin1 and LC3-II/LC3-I ratio increased and protein p62 decreased with the treatment of rapamycin when compared with unstimulated control. A reversion of protein expression mentioned above was observed after the pretreatment of hBD3 at the concentration of 5 μg/ml for 12 h (Figs. 2A and 3B). The expression of p62 was also decreased by immunofluorescence, which was accordant with western blotting (Fig. 2B). Ad-mRFP-GFP-LC3 was used to monitor autophagic flux based on the different pH stabilities of mRFP and GFP, as the GFP signal would be quenched by the acidic environment of the lysosome. Therefore, autophagosomes were represented by the yellow merged dots (mRFP^+^ GFP^+^), while the autophagic flux indicated by the formation of autolysosomes was reflected by the red dots (mRFP^+^ GFP^−^). Our results demonstrated a significant decrease of mRFP-GFP colocalization dots and free red dots after hBD3 treatment compared with the autophagy induction group, confirming its inhibition of autophagic flux in IEC-6 cells (Fig. 2C–E). Transmission electron microscopy (TEM) was used to monitor the ultrastructure of IEC-6 and Caco2 cells. After the rapamycin exposure, degradative autophagic vacuoles (AVd) were markedly accumulated, while hBD3 treatment could decrease the formation of AVd (Fig. 2F). Taken together, hBD3 treatment could inhibit excessive autophagy in cultured intestinal epithelial cell.

### Involvement of CXCR4 in hBD3-mediated autophagy suppression

It has been reported that CXCR4 was involved in mTOR-dependent migration of gastric carcinoma cells^17^. Moreover, structural analysis revealed similarities between hBD3 and SDF-1α, natural ligand of CXCR4^18,19^. In order to confirm the role of CXCR4 signaling pathway in the process of hBD3-mediated autophagy suppression in enterocytes, CXCR4 inhibitor (AMD3100) and specific CXCR4 small interfering RNA (siRNA) were used to pretreat the intestinal epithelial cells. Compared with hBD3 treatment group, the autophagy-associated proteins Beclin1 and LC3-II/LC3-I ratio increased after incubation with 10 uM AMD3100 for 24 h, while protein p62, p-AKT and p-mTOR were showed in decline expression (Fig. 3A,B). Moreover, IEC-6 and Caco2 cells were transfected with CXCR4 siRNA to further confirm the results caused by CXCR4 inhibition. The knockdown efficiency of CXCR4 siRNA was approximately 60% and resulted in a remarkable reduction in CXCR4 expression compared with control siRNA (Fig. 3C,D). After being transfected with CXCR4 siRNA for 36 h, the expression of autophagy-associated proteins was evaluated and the results showed that the effects on protein expression induced by transfection of CXCR4 siRNA were more significant than that of inhibitor AMD3100 (Fig. 3E,F). Together, these findings indicated that the CXCR4 might be involved in the hBD3-induced autophagy inhibition.

### hBD3-mediated autophagy suppression attenuated the inhibition of intestinal epithelial cell migration

**Figure 4:**
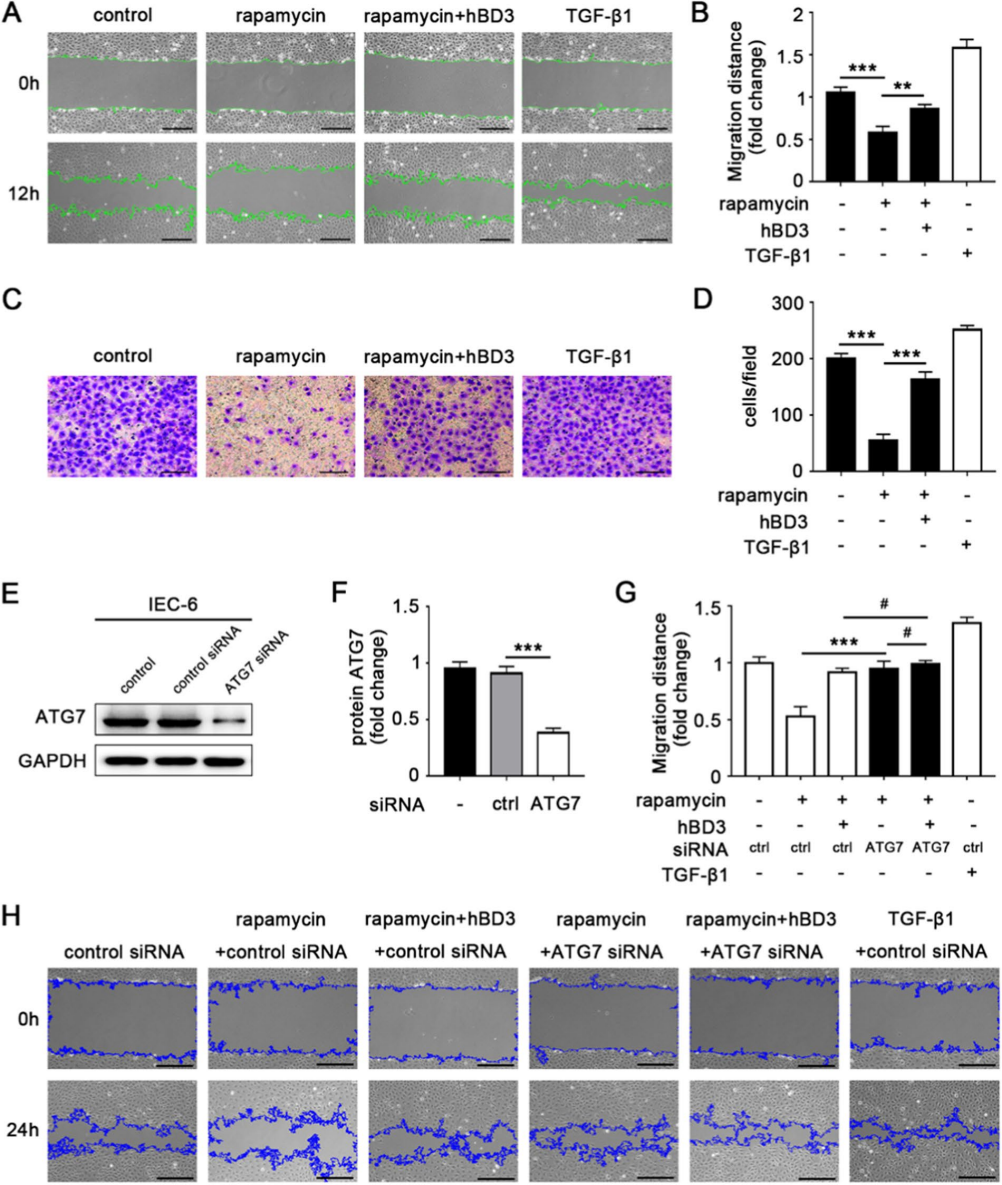
hBD3-mediated autophagy suppression promoted intestinal epithelial cells migration. Migration ability of IEC-6 was determined by wound healing assay (**A**, scale bar, 200 μm) and transwell migration assay (**C**, scale bar, 200 μm), and then analyzed respectively (**B**,**D**). ATG7 knockdown efficiency of IEC-6 cells was assessed by western blotting (**E**) and evaluated detailedly. (**F**) Migration ability of IEC-6 transfected with ATG7 siRNA was detected by wound healing assay (**H**, scale bar, 200 μm) and subsequently compared with other groups (**G**) to confirm the effect of hBD3-mediated autophagy suppression on the migration of intestinal epithelial cells. Data was expressed as mean ± SD from three independent experiments and representative images were presented. **p < 0.01, ***p < 0.001, ^#^p > 0.05.

Decreased mucosal barrier repairing capacity in neonates, especially in premature infants, is one major risk factor of necrotizing enterocolitis. Therefore, wound healing assay and transwell migration assay were conducted to measure the migration ability of IEC-6 cells in different groups. In our study, the migration distance of IEC-6 cells was significantly decreased in the group incubated with rapamycin compared with the unstimulated control. However, pretreatment of hBD3 could noticeably reverse the inhibition effect of rapamycin (Fig. 4A,B). The results of the transwell migration assay were consistent with the wound healing assay with the TGF-β1 as the positive control (Fig. 4C,D). IEC-6 cells were transfected with ATG7 siRNA to confirm the effect of hBD3-mediated autophagy suppression on the migration of intestinal epithelial cells. Knockdown efficiency of ATG7 siRNA was more than 65% and markedly down-regulated the protein expression of ATG7 compared with control siRNA (Fig. 4E,F). The migration distance of IEC-6 cells transfected with ATG7 siRNA increased obviously compared with autophagy induction group no matter whether hBD3 incubation or not, which indicated that hBD3 did regulate intestinal epithelial cell migration by inhibiting autophagy (Fig. 4G,H).

**Figure 5:**
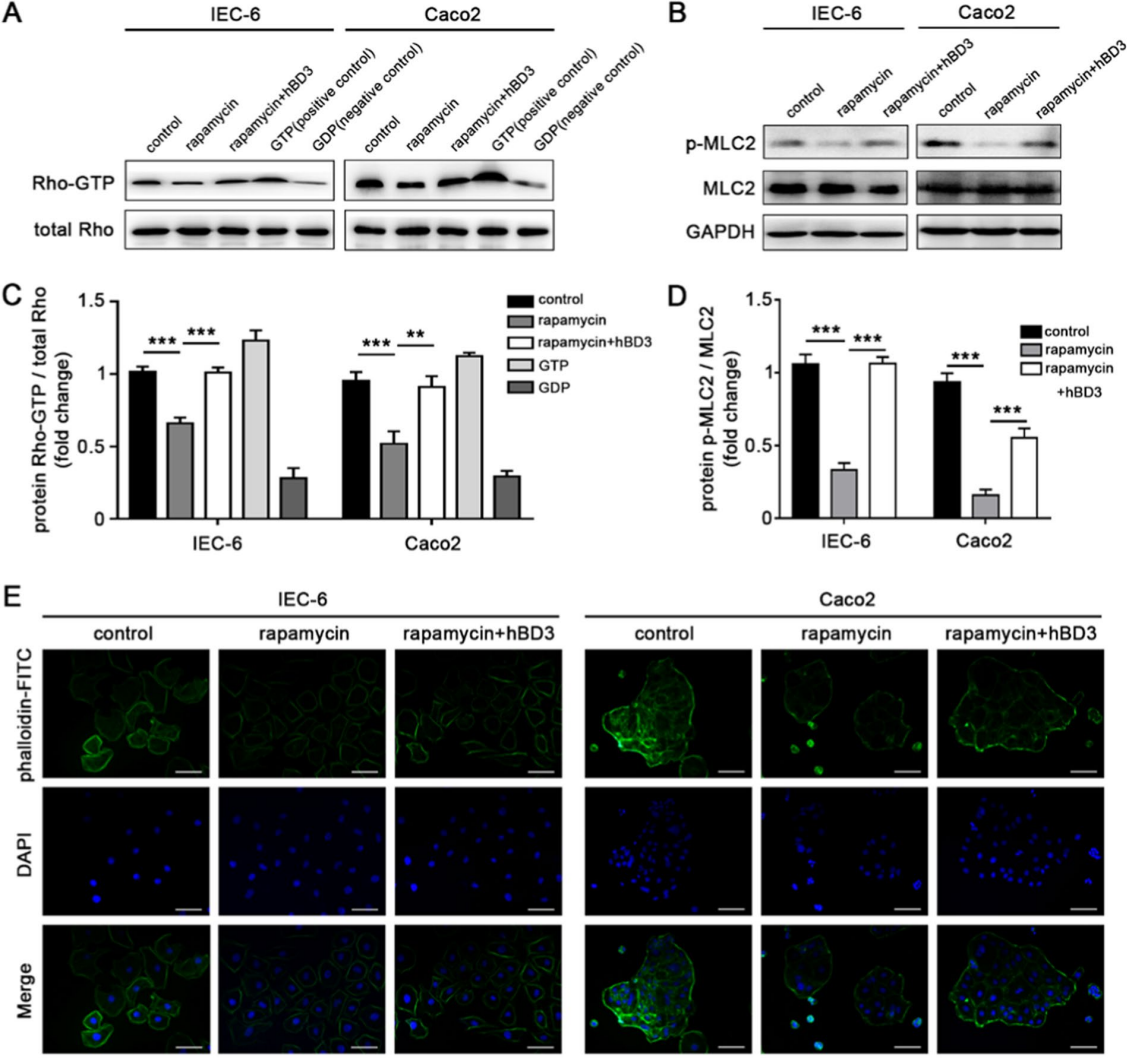
hBD3 promoted intestinal epithelial cells migration via the Rho signaling pathway. Rho-GTP was extracted by pull-down assay and its expression levels in different groups were measured by western blotting (**A**) with the total Rho as an input control. GTP and GDP were respectively added to act as the positive and negative control. Relative Rho-GTP expression in each group was quantified. (**C**) The expression of p-MLC2 in each group was assessed by western blotting (**B**) with the total MLC2 as a loading control. Relative p-MLC2 expression in different groups were compared. (**D**) F-actin was stained with phalloidin- FITC and analyzed by fluorescence microscopy (**E**, scale bar, 50 μm). Data was expressed as mean ± SD from three independent experiments and representative images were presented. Abbreviations: GTP: guanosine triphosphate; GDP: guanosine diphosphate; MLC2: myosin light chain-2; FITC: fluorescein isothiocyanate. **p < 0.01, ***p < 0.001.

It has been reported that the Rho signaling pathway played an important role in the regulation of cell migration^20^. Thus, we first examined the protein expression of activated Rho (Rho-GTP) with GTP as the positive control and GDP as the negative control. Autophagy induced by rapamycin down-regulated the activation of Rho, while hBD3 markedly increased the conversion from Rho-GDP to Rho-GTP (Fig. 5A,C). In addition, the downstream effectors were also assessed. Phosphorylation of Myosin Light Chain 2 (MLC2) was detected by western blotting which demonstrated that hBD3 up-regulated the phosphorylation of MLC2 compared with the autophagy induction group (Fig. 5B,D). The accumulation of F-actin in IEC-6 and Caco2 cells incubated with fluorescein isothiocyanate labeled phalloidin was monitored. hBD3 could effectively attenuate the inhibition effect of rapamycin (Fig. 5E).

Taken together, we concluded that autophagy inhibited the migration of intestinal epithelial cells and hBD3 treatment could significantly mitigate the inhibition effect induced by autophagy, which was confirmed to be associated with the Rho activation, MLC2 phosphorylation and F-actin accumulation.

### Preliminary evaluation of hBD3-mediated autophagy inhibition in rat NEC model

**Figure 6:**
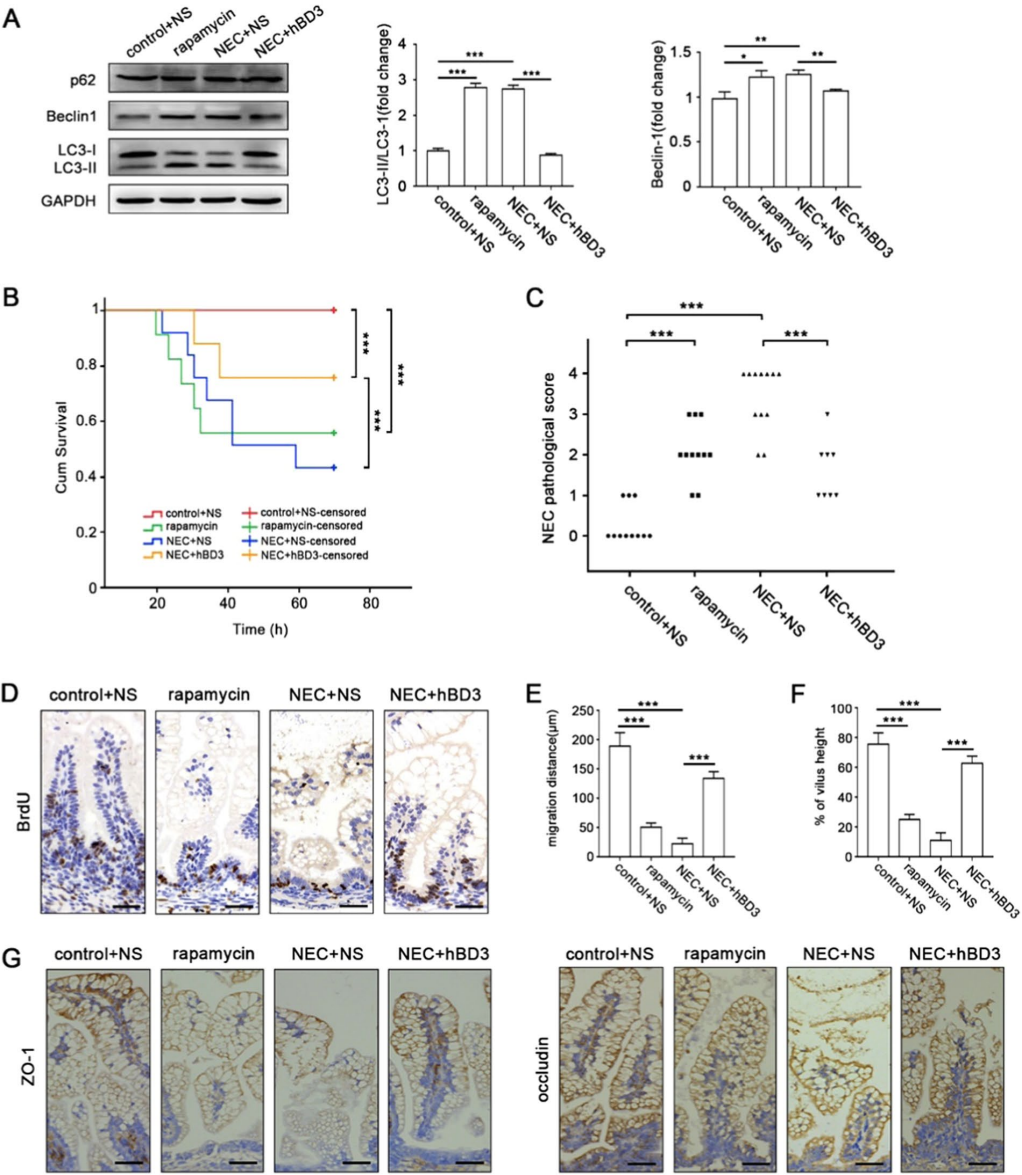
Preliminary evaluation of hBD3-mediated autophagy inhibition in rat NEC model. The expression of autophagy-related proteins LC3, p62 and Beclin1 in the ileum of rats among four groups was assessed by western blotting, the LC3II/LC3I ratio and Beclin1 expression was analyzed. (**A**) Kaplan Meier survival analysis was used to identify the differences of survival time in groups. (**B**) NEC pathological score was conducted in each group by a pathologist independently and compared with other groups as indicated. (**C**) Rats were intraperitoneal injected with BrdU 18 h before killing and immunostained with BrdU antibody to measure the enterocyte migration (**D**, scale bar, 50 μm). The migration distance (**E**) and mobility (**F**) were evaluated. ZO-1 and occludin were immunostained on the sections of the terminal ileum from rats among different groups (**G**, scale bar, 50 μm). Data was expressed as mean ± SD from three independent experiments and representative images were presented. Abbreviations: Abbreviations: BrdU: 5-Bromo-2′-deoxyuridine, NS: Normal Saline. *p < 0.05, **p < 0.01, ***p < 0.001.

Sprague–Dawley neonatal rats were divided randomly into four groups: Control + NS, rapamycin, NEC + NS and NEC + hBD3. The rat NEC model was further optimized in our study, including hypertonic formula feeding combined with asphyxia-cold stress exposure^15,21,22^, to better stimulate clinical development of NEC. The effect of hBD3 on autophagy in the ileum was assessed first. The conversion of LC3-I to LC3-II and protein expression of Beclin1 were significantly increased in group rapamycin and group NEC + NS, while the protein expression of p62 was unchanged significantly. Autophagic activity was down-regulated after hBD3 treatment (Fig. 6A). The results of our study showed that body weight of the formula-fed rats was markedly lower than that of the mother-fed rats. Rats in group NEC suffered obvious weight loss, however, the body weight increased to some extent after hBD3 intervention and the difference was statistically significant (P = 0.007, Supplemental Fig. 1 and Table S1). The survival rate of rats was significantly decreased from 100% in control group to 42% in NEC group, which could be effectively improved by the treatment of hBD3 (P < 0.001, Fig. 6B and Table S2). All pups were sacrificed and the entire digestive tract was displayed to present the macroscopic changes among different groups. Edema and necrosis of the small intestine was observed in group NEC, which could be effectively ameliorated with the administration of hBD3 (Supplemental Fig. 2). Hematoxylin and eosin (H&E) staining of terminal ileum tissue was performed and representative images were shown (Supplemental Fig. 2). The degree of bowel damage, evaluated by the histological scores of ileum, was reduced in group NEC + hBD3, compared with the group NEC, with a mean NEC score of 1.33 and 2.52, respectively (P < 0.001, Fig. 6C).

Rats in each group were intra-peritoneal injected with BrdU 18 h before sacrificed. The migration distance was indicated by the distance between the bottom of the small intestine crypt to the apical cell labelled BrdU, and the mobility were evaluated by the ratio of migration distance to intestinal villus height. Representative images were shown and the intestinal epithelial cells migration ability was markedly decreased in group rapamycin and in group NEC, which was effectively up-regulated after hBD3 administration (Fig. 6D–F). Inflammation played a central role in the development of NEC, we detected the inflammatory cytokine expression in ileum and in serum to further explore the underlying relationship between hBD3-mediated autophagy inhibition and inflammatory cytokine expression. In group NEC, inflammatory cytokines including IL-6, IL-10 and TNF-α were noticeably increased in the ileum and serum, but the intervention of hBD3 could relatively down-regulated the indicated cytokines (Supplemental Fig. 3) and then attenuated the inflammatory injury of rat NEC model. ZO-1 and occludin were immunostained to evaluate the mucosal integrity. ZO-1 staining loss was observed in group NEC, which was consistent with occludin staining. The inhibition of ZO-1 and occludin expression could be prevented to some extent after hBD3 intervention (Fig. 6G).

Together, hBD3-stimulated autophagy down-regulation promoted intestinal epithelial cells migration and mucosal integrity, and down-regulated inflammatory cytokines expression.

## Discussion

In the present study, we reported that initiation of autophagy was blocked in cultured intestinal epithelial cells after hBD3 treatment. Furthermore, we provided evidence that impaired enterocyte migration was ameliorated both in enterocytes and in a rat model of NEC by hBD3, which was associated with inhibition of excessive intestinal autophagy. Our data suggested an additional role of hBD3-mediated protection against intestinal mucosal injury: inhibition of over-activated autophagy in small intestine.

Previous studies^23–25^ have demonstrated that G-protein coupled receptor CXCR4 was widely expressed in intestinal epithelial cells. And activation of CXCR4, by its natural ligand SDF-1α, was involved in migration of enterocytes and restitution of wounded barrier. In current study, we did not provide direct evidence of hBD3 binding to CXCR4. However, similarities indicated by structural analysis between hBD3 and SDF-1α, including the β-sheet conformation and three N-terminal surface cationic residues, may be the molecular structure basis of their binding to CXCR4. The β-sheet conformation is stabilized by three intramolecular disulfide bonds among six conserved cysteines^18^. Three N-terminal surface cationic residues (i.e., K1, R8 and R12) are believed to be the key structure of CXCR4-SDF-1α binding^19^. Correspondingly, three similar cationic residues are present on the surface of hBD3: K8, K32, and R36. Substituting all six cysteines or all three cationic residues of hBD3 was reported to result in an obviously decreased combination with CXCR4^26^. Thus, we speculated that the distinctive conformation was essential for hBD3-interaction with CXCR4.

Autophagy can be activated by stress such as starvation and injury to maintain cell survival by degrading harmful proteins and damaged organelles^27–29^. The process of autophagy can be divided into four stages: the formation of the separation membrane, the formation of autophagosomes, the fusion of autophagosomes and lysosomes, and the degradation of autophagolysosomes^30^. Different methods were applicated to evaluate the autophagy level according to the guideline. The expression of autophagy-related proteins, Beclin1 and LC3-II/LC3-I ratio, noticeably increased both in rapamycin treated cells and in the rat NEC model, while the protein expression of p62 in ileum tissues was statistically insignificantly among different rat groups. One possible explanation is that p62 is a multifunctional protein regulated by a variety of signaling pathways, including NF-κB signaling pathway, and apoptosis pathway^31–33^. Because of just using of rapamycin in current study, only initiation of autophagy was evaluated after hBD3 treatment. Reduction in degradative activity as a result of a block in trafficking to lysosomes might also be involved in the process.

The destruction of mucosal integrity in neonates, especially in premature infants, is one critical cause of NEC. Intestinal epithelial cell migration plays a vital role in the reparative process of mucosal barrier. Cell migration occurs within a few hours after injury with intestinal epithelial cells losing polarity and migrating to the lesion through epithelial-mesenchymal transition. In children with NEC, it has been proved that the expression and distribution of genes involved in the reparative process of intestinal mucosal barrier were abnormal^34^. Recent research has revealed that autophagy induced by rapamycin noticeably inhibits the migration of intestinal epithelial cells^11^. Our results showed that impaired enterocyte migration was induced after activation of autophagy, and hBD3 treatment promoted the migration of intestinal epithelial cells. In the early stage of autophagosome formation, the Atg5-Atg12 complex binds to LC3-PE complexes via the kinase Atg7 and then promotes the localization of LC3-PE on autophagosomes^35^. Thus, ATG7 is an essential part of the whole autophagy process. ATG7 siRNA was transfected in our study to confirm that the promotion effect of hBD3 on intestinal epithelial cell migration was mediated by inhibiting autophagy. The activation of Rho, a GTPase in the Ras superfamily, and its downstream effectors is essential in the regulation of cell migration^20^. Our data demonstrated that hBD3-mediated autophagy inhibition could significantly promote cell migration by activating Rho protein, phosphorylating MLC2 and accumulating F-actin, resulting in intestinal mucosal healing. Obviously, there are other molecular mechanism underlying hBD3-mediated protection. For instance, recent studies suggested defensin also serves to help shape the composition of the intestinal microbiota^36^. And the effects of hBD3 on these targets deserve further investigation.

In summary, we reveled that down-regulation of excessive autophagy might be involved in hBD3-mediated protection, which resulted in the promotion of enterocyte migration and reduction of the severity of NEC in rat pup model. These results also provide evidence of therapeutic potential of host defence peptides.

## Materials and Methods

### Reagents and antibodies

Human β-defensin-3 (hBD3) was purchased from PEPTIDE INSTITUTE, INC (#4382-s, Ibaraki, Osaka, Japan). Reagents like rapamycin (#V900930), Lipopolysaccharides (LPS, #L4524), Chloroquine (CQ, #C6628), 5-Bromo-2′-deoxyuridine (BrdU, #B5002) were acquired from Sigma (St. Louis, MO, USA). Plerixafor (AMD3100) was procured from Selleckchem (#S8030, Houston, TX, USA). Reagents like Dulbecco’s modified eagle medium (DMEM) (#11995065), Fetal bovine serum (FBS) (#10099141), Opti-MEM® Reduced-Serum Medium (#31985062), 0.25% Trypsin-EDTA (#25200072), Penicillin Streptomycin (#15140122), Phosphate Buffered Saline (PBS) (#10010023) were obtained from Gibco (Grand Island, NY, USA). Antibodies against CXCR4 (#ab124824), p-AKT (Phospho- T308) (#ab38449), AKT (#ab8805), Beclin1(#ab207612), SQSTM1/P62 (#ab109012) were from Abcam (Cambridge, UK). Other antibodies, including anti- LC3A/B (#4108S), mTOR (#2972S), p-mTOR (#2971S), Myosin Light Chain 2 (MLC2, #8505T), p-Myosin Light Chain 2 (S19) (#3671T), FAK (#13009T), p-FAK (Y576/577) (#3281T), Glyceraldehyde-3-phosphate dehydrogenase (GAPDH, #5174S) were purchased from Cell Signaling Technology (CST, Beverly, MA, USA).

### Cell culture

Rat intestinal crypt epithelial cell line IEC-6 and human colorectal adenocarcinoma cell line Caco-2, provided by the Cell Resource Center of the Chinese Academy of Science (Shanghai, China), were cultured in DMEM supplemented with 10% FBS and 1% Penicillin Streptomycin solution at 37 °C with humidified 5% CO_2_ atmosphere.

### Protein extraction and western blotting

Western blotting was performed as described previously^15,22^. Cells were treated as required in each group and then lysed step by step in RIPA lysis buffer containing 1 mM phenylmethyl sulfonylfluoride (PMSF) (CST, #8553S, Beverly, MA, USA) and 1% protease inhibitor cocktail (Sigma, #P8340, St. Louis, MO, USA). The lysate (30 ug) was separated by 10% or 12% SDS-PAGE and transferred to PVDF membranes (Millipore, ISEQ. 00010, Billerica, MA, USA). Afterwards, the membrane was incubated with blocking buffer (TBS containing 0.1% Tween-20 and 5% BSA) at room temperature for 1 h. Subsequently, indicated primary antibodies were used to probe the membrane with gentle agitation at 4 °C overnight. The membrane was then incubated with Peroxidase-conjugated AffiniPure Goat Anti-Rabbit IgG (H + L) (Jackson ImmunoResearch, #111-035-003, West Grove, PA, USA). The result of western blotting was detected by Immobilon^TM^ Western Chemiluminescent HRP Substrate (Millipore, #WBKLS0500, Billerica, MA, USA).

### Transmission electron microscopy (TEM)

Morphology of autophagic structures during the dynamic maturation process from phagophore through autolysosome was revealed by the transmission electron microscopy (TEM) (HITACHI, HT7700, Chiyoda, Tokyo, Japan). First, cells in each group were collected by cell scrapers (CORNING, #3010, Corning, NY, USA) and then fixed in 2.5% glutaraldehyde after centrifugation at 800 rpm for 5 min. Afterwards, cells were post-fixed with 1% Osmium tetroxide (OsO_4_) in 0.1 M PBS for 2 h at room temperature. Dehydration, infiltration, embedment and cutting of ultrathin sections was carried out subsequently. After staining with uranyl acetate and lead citrate for 15 min respectively, ultrathin sections were observed and photographed with TEM.

### RNA extraction and quantitative real-time PCR (qRT-PCR)

TRIzol^TM^ reagent (Invitrogen, #15596026, Waltham, MA, USA) was used to extract total RNA of rat ileum tissues in each group according to the instruction of the manufacturer. Afterwards, cDNA was synthesized by PrimeScript^TM^ RT Master Mix (Takara, #RR036A, Kusatsu, Shiga, Japan). Subsequently, FastStart Essential DNA Green Master (Roche Diagnostics, #06924204001, Indianapolis, IN, USA) was used to monitor the target gene amplification dynamically. Primer sequences are listed in Table S3. The relative expression of mRNA was calculated with the 2^−ΔΔCt^ method.

### Small interfering RNA (siRNA) transfection

Small interfering RNA (siRNA) products respectively targeting at CXCR4 and ATG7 were procured from QIAGEN (Duesseldorf, Germany). Cells were seeded into 6-well culture plates at a final concentration of 2 × 10^5^ cells/ml in DMEM and then incubated in the incubator for the short time until transfection. A mixture supplemented with 800 ng siRNA, 100 ul Opti-MEM^®^ reduced-serum medium and 24 ul HiperFect Transfection Reagent (QIAGEN, #301705, Duesseldorf, Germany) were blended and incubated at room temperature for 10 min to allow the formation of transfection complexes. Complexes were drop-wised onto the cells and then incubated in the incubator. The efficiency of gene silencing was monitored by western blotting using validated antibodies 36 h after transfection. The effect of CXCR4 knockdown on hBD3 mediated suppression of autophagy was assessed with the expression levels of autophagy associated proteins by western blotting. The effect of ATG7 knockdown on autophagy mediated retardation of cell migration was assessed with wound healing assay.

### Immunofluorescence

The cells were seeded on the coverslips disinfected and sterilized thoroughly at a final concentration of 1 × 10^5^ cells/ml in DMEM. When cells reached 50% confluence, following treatments in each group were performed at the indicated time points. The cells were then rinsed with PBS for 3 times and fixed with 4% paraformaldehyde for 30 min, followed by soaking the cells in absolute ethyl alcohol for 20 min. After being blocked with blocking buffer contained with 1% BSA, 4% normal serum, 0.4% TritonX100 and 95.6% PBS for 30 min, the cells were subsequently stained with the indicated primary antibodies at 4 °C overnight. Then, the cells were incubated with CyTM3-conjugated AffiniPure Donkey Anti-Rabbit IgG (H + L) (Jackson ImmunoResearch, #711-165-152, West Grove, PA, USA) for 1 h at room temperature away from light after washing the nonspecific binding antibody with PBT supplemented with 99.9% PBS and 0.1% TritonX100. Finally, the nuclei were labeled with 4,6-diamidino-2-phenylindole (DAPI, #32670, Sigma, St. Louis, MO, USA) for 5 min at room temperature, and then the cells were rinsed with PBS for 3 times. The stained cells in each group were observed and photographed with the fluorescent microscope (Leica, Wetzlar, Germany).

### Adenovirus infection and laser confocal detection

Ad-mRFP-GTP-LC3 (HANBIO, #HB-AP2100001, Shanghai, China) was designed to monitor the induction of autophagy and the autophagy flux through detecting the degradation of GTP signal in the acidic condition of the lysosome lumen. Cells were seeded into 12-well culture plates at a final concentration of 1 × 10^5^ cells/ml in DMEM. When cells reached 30% confluence, Ad-mRFP-GTP-LC3 (1.26 × 10^10^ PFU/ml) was added into each well and the multiplicity of infection (MOI) was 1000 following the manufacturer’s instructions. Following treatments in each group were performed 48 h after infection. After being rinsed with PBS for 3 times and fixed with 4% paraformaldehyde for 30 min, the cells were observed and photographed with the confocal microscopy (NIKON ECLIPSE TI, Chiyoda-KU, Tokyo, Japan).

### Wound healing assay

Cell migration was assessed by wound healing assay. The horizontal lines were evenly drawn on the back of the 6-well culture plate with 0.5 cm spacing. Cells were seeded into the 6-well culture plate at a final concentration of 5 × 10^5^ cells/ml in DMEM. When cells reached 100% confluence, sterile micropipette tips were used to scratch straight lines which were perpendicular to the horizontal lines on the back of the 6-well culture plate on the confluent monolayer cells. The interactions were used to mark the same field of microscope so that the dynamical changes of cell migration could be accurately measured by photographing five designated interactions at different indicated time points. The cell migration results were analyzed by ImageJ software.

### Transwell migration assay

The transwell migration assay was a classical technique to quantify cell movement. 100 μl cell suspension containing 1 × 10^5^ cells in Opti-MEM® Reduced-serum Medium was placed on the upper layer of the transwell chamber with 8 μm permeable membrane, and 500 μl DMEM containing 10% FBS was placed below the cell permeable membrane. Following an incubation period for 24 h, the transwell chamber was fixed by 4% paraformaldehyde for 30 min, and then the cells remaining on the upper layer of the permeable membrane were scraped with cotton swabs. After staining with 0.1% crystal violet for 15 min, the amount of migration cells in the bottom of the chamber was counted randomly in five microscopic fields.

### Active Rho and downstream effectors detection

The Active Rho Detection Kit (CST, #11860S, Beverly, MA, USA) was used to detect the Rho activation level following the manufacturer’s protocol. The whole pull-down process included the binding of GTP-bound GTPase and glutathione resin through GST-linked binding protein, the centrifugation to remove unbound proteins, and the elution to remove glutathione resin through SDS buffer. Then, the elute sample could be analyzed by western blotting.

The downstream effector included Myosin Light Chain 2 (MLC2) and F-actin. MLC2 phosphorylation (Ser19) was detected by western blotting. To detect cellular F-actin expression, the cells were seeded into 12-well culture plate at a final concentration of 1 × 10^5^ cells/ml in DMEM and incubated overnight, following treatments in each group were performed at the indicated time points. When cells reached 50% confluence, rinse, fixation and permeabilization were performed step by step, and then the cells were incubated with 50 g/ml fluorescein isothiocyanate labeled phalloidin for 30 min at room temperature away from light. The fluorescent microscope was used to observe and photograph the stained cells in each group.

### Serum inflammatory factors measure

Enzyme linked immunosorbent assay (Elisa) was used to measure the expression levels of the inflammatory factors in serum. Elisa Kits targeting at TNF-α, IL-6 and IL-10 were obtained from ELK Biotechnology (#ELK1396, #ELK1158, #ELK1144, Wuhan, China). After clotting for two hours at room temperature, the serum was centrifuged for 20 min at approximately 1000 × g. Freshly prepared serum was used to detect inflammatory factors with the detailed manufacturer’s instructions. The microplates were pre-coated with an indicated antibody specific to the inflammatory factor, and the color change caused by the enzyme-substrate reaction could be measured spectrophotometrically at a wavelength of 450 nm.

### Animal experimental design and evaluation

Experimental design was approved by the Animal Care Committee of the Children’s Hospital of Shanghai. It is confirmed that all methods were performed in accordance with the relevant guidelines and regulations. The rat NEC model described by Caplan *et al*.^21^ was used for reference and further optimized to better simulate the occurrence and development of NEC. Hypertonic formula feeding and asphyxia-cold stress exposure were conducted in our rat NEC model^15,22^. Forty-two newborn Sprague–Dawley rats originated from six different litters were divided randomly into four groups.

Control + NS (n = 11), Control + rapamycin (n = 11), NEC + NS (n = 12), and NEC + hBD3 (n = 8). 18 h before the end of the experiment, BrdU (50 mg/kg) was intra-peritoneal injected to assess the migration of intestinal epithelial cells. All pups were sacrificed via cervical dislocation and the intestine and serum were collected according to demand. Hematoxylin and eosin (H&E) staining was used for experienced pathologists to score the pathological changes blindly according to the published NEC scoring system. Intestine tissues with scores 2 or higher were defined as NEC positive. Autophagy related proteins were assessed by western blotting. Inflammatory cytokines were measured via qRT-PCR and Elisa respectively. Tight junction proteins and BrdU were detected with immunohistochemistry.

### Statistical analysis

Statistical analysis was performed with SPSS 24 software (SPSS, Chicago, Illinois, USA). Results were represented as Mean ± SD. Student’s t test and one-way analysis of variance (ANOVA) were used for comparison among groups. P < 0.05 was considered as statistically significant.

## Supplementary information

Supplementary information
