# Supplementary material for: Human β-defensin-3 reduces excessive autophagy in intestinal epithelial cells and in experimental necrotizing enterocolitis
Source: Sci Rep. 2019 Dec 27;9:19890. doi: 10.1038/s41598-019-56535-3 (PMC6934505; doi:10.1038/s41598-019-56535-3)
Supplement: Supplementary file 1 — Supplementary information [file 41598_2019_56535_MOESM1_ESM.docx]

**Human β-defensin-3 reduces excessive autophagy in intestinal epithelial cells and in experimental necrotizing enterocolitis**

Liping Chen, Zhibao Lv, Zhimei Gao, Guijie Ge, Xueli Wang, Junmei Zhou, Qingfeng Sheng

**Supplemental Figure 1**


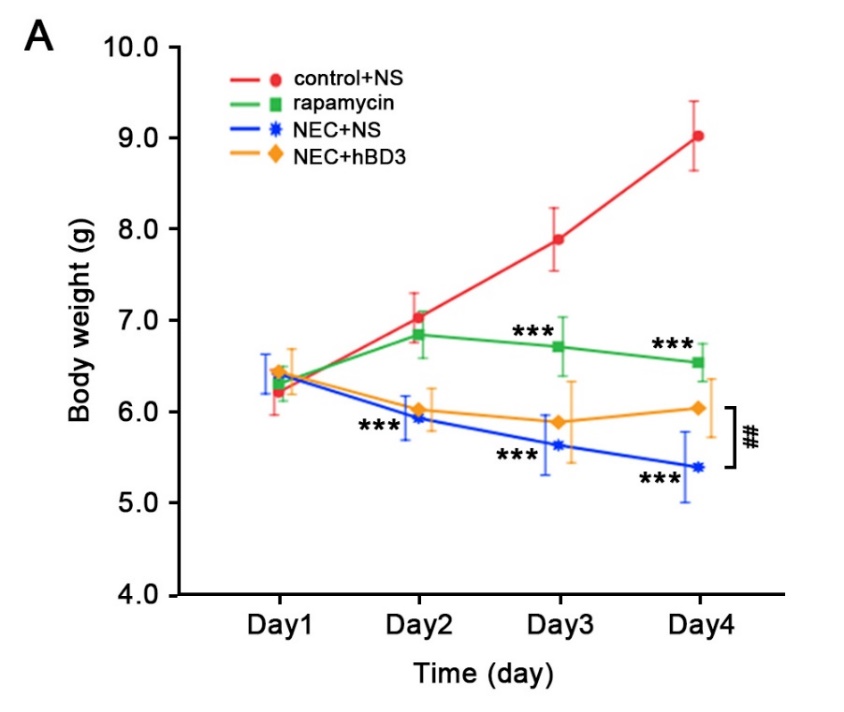


**Body weight of rats among groups was detailedly recorded and analyzed** (**A**). Rats in group NEC+NS suffered obvious weight loss, which could be significantly attenuated to some extent after hBD3 administration (p=0.007). ***p<0.001 as compared between rapamycin and NEC with control; ^##^ p<0.01 as compared between NEC+hBD3 with NEC.

**Supplemental Figure 2**


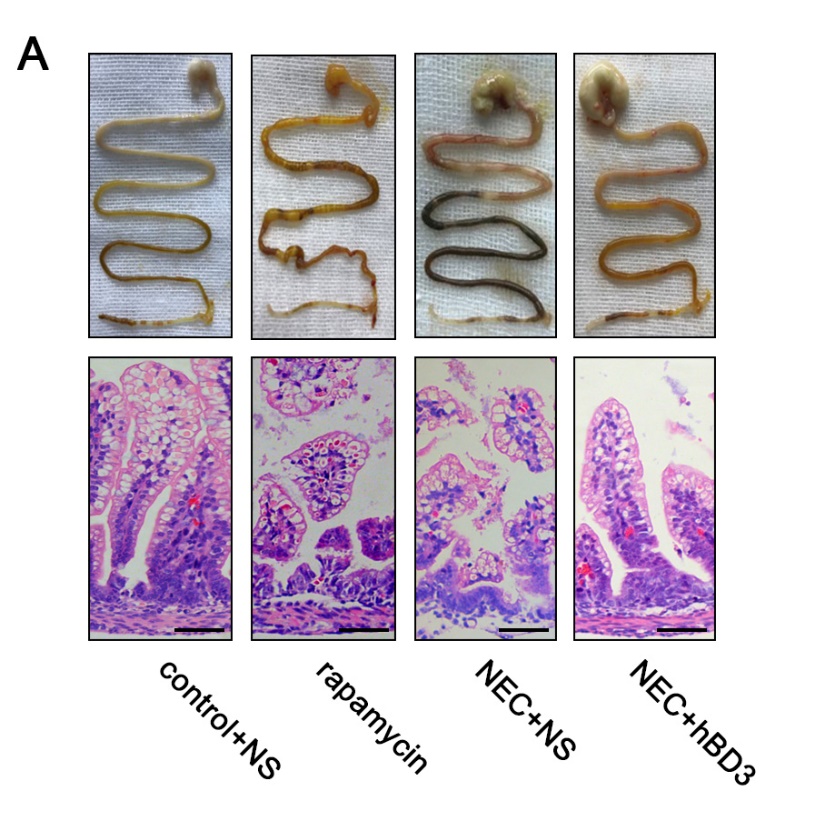


**Macroscopic changes of the entire digestive tract and representative Hematoxylin and eosin (H&E)-stained images of rats distant ileum among groups were shown (A).** Edema and necrosis of the small intestine and the disorder of intestinal epithelial structure were observed in NEC+NS and rapamycin, however, hBD3 intervention could ameliorate the degree of bowel damage.

**Supplemental Figure 3**


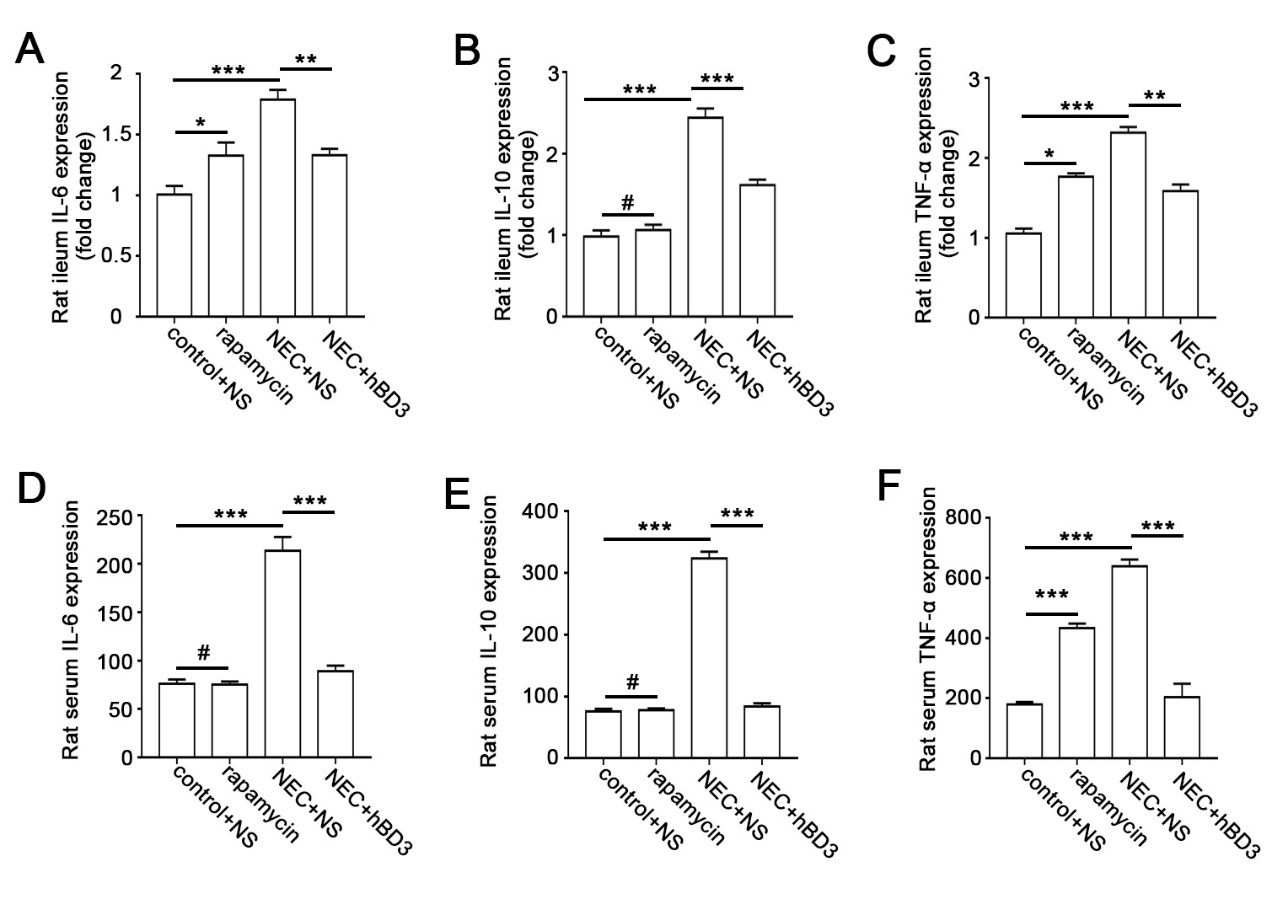


**The expression of inflammatory cytokines both in the ileum tissues (A, B and C) and serum (D, E and F).** The expression of inflammatory cytokines including IL-6, IL-10 and TNF-α was respectively calculated and were noticeably increased in group NEC+NS, while the intervention of hBD3 could relatively down-regulated the indicated cytokines. Abbreviations: IL-1:interleukin, TNF-α: tumor necrosis factor-α. *p<0.05, **p<0.01, ***p<0.001, #p>0.05

**Supplemental Table 1. Body weight(g) of neonatal rats**

| Group | Day1 | Day2 | Day3 | Day4 |
| --- | --- | --- | --- | --- |
| control+NS | 6.12±0.43 | 6.97±0.46 | 7.85±0.89 | 9.02±0.65 |
| Rapamycin | 6.22±0.29 | 6.78±0.39 | 6.64±0.40*** | 6.46±0.20*** |
| NEC+NS | 6.33±0.35 | 5.83±0.37*** | 5.53±0.32*** | 5.28±0.32*** |
| NEC+hBD3 | 6.36±0.31 | 5.93±0.29 | 5.79±0.44 | 5.95±0.31**^##^** |

Data are presented as mean ± SD.

NS, normal saline; NEC, necrotizing enterocolitis.

***Statistical significance with P < 0.001 (Rapamycin and NEC+ NS vs. control+NS).

**^##^**Statistical significance with P < 0.01 (NEC+NS vs. NEC+ hBD3).

**Supplemental Table 2. Survival rate and NEC score of neonatal rats**

| Group | Survival rate | Pathological score  (mean ± SD) |
| --- | --- | --- |
| control+NS | 100% | 0.15±0.38 |
| Rapamycin | 55%*** | 2.11±0.89*** |
| NEC+NS | 42%*** | 2.52±0.84*** |
| NEC+hBD3 | 75%**^###^** | 1.33±0.52^###^ |

NS, normal saline; NEC, necrotizing enterocolitis.

***Statistical significance with P < 0.001 (Rapamycin and NEC+ NS vs. control+NS).

**^###^**Statistical significance with P < 0.001 (NEC+NS vs. NEC+ hBD3).

| Target gene | Primer sequences |
| --- | --- |
| IL-6 | (F)5’-TGCCTTCTTGGGACTGAT-3’ |
|  | (R)5’-ACTGGTCTGTTGTGGGTG-3’ |
| TNF-α | (F)5’-CTGGCGTGTTCATCCGTTCTCTAC-3’ |
|  | (R)5’-GATCCACTCAGGCATCGACATTCC-3’ |
| IL-10 | (F)5’-CTGCTCTTACTGGCTGGAGTGAAG-3’ |
|  | (R)5’-CACCTGCTCCACTGCCTTGC-3’ |
| actin | (F)5’-TGCCGCATCCTCTTCCTC-3’ |
|  | (R)5’-GGTCTTTACGGATGTCAACG-3’ |

**Supplemental Table 3. Sequences of oligonucleotide primers**

IL, interleukin; TNF-α, tumor necrosis factor-α.
